# 

**Published:** 1889-12

**Authors:** 


					﻿io cts. a copy
DECEMBER 1883.
$ i .00 per year.
HALLS «
gJOVRNAL^f
HEAlT El
ESTABLISHED 1854
l/°t- 36
W°.- 12
OFFICE; 206 BROADWAY. EVENING- POST BUILDING. Room 11. N. Y.
Entered as Second-class Matter at the Post-Office, New York,
				

